# The role of endoplasmic reticulum aminopeptidase ERAP2 pathogenic mutation rs1363907 in amoxicillin clavulanate-induced liver injury: a special case report

**DOI:** 10.3389/fphar.2025.1564124

**Published:** 2025-05-21

**Authors:** Yan Zhan, Jin-Mao Liao, Ling Ye, Long Zhang, Zheng Zhang, Ji-Ye Yin

**Affiliations:** ^1^ Department of Clinical Pharmacology, Xiangya Hospital, Central South University, Changsha, China; ^2^ Hunan Key Laboratory of Pharmacogenetics, Institute of Clinical Pharmacology, Central South University, Changsha, China; ^3^ Engineering Research Center of Applied Technology of Pharmacogenomics, Ministry of Education, Changsha, China; ^4^ National Clinical Research Center for Geriatric Disorders, Changsha, Hunan, China; ^5^ Department of Hepotology, Hunan Provincial People’s Hospital, The First Affiliated Hospital of Hunan Normal University, Changsha, China; ^6^ Hunan Jiarun Medical Laboratory Co., Ltd., Changsha, Hunan, China

**Keywords:** AC-DILI, idiosyncratic hepatotoxicity, ERAP2, HLA polymorphism, autoimmune phenomena

## Abstract

Idiosyncratic hepatotoxicity is a type of drug-induced liver injury (DILI) that is unpredictable and clinically severe, and amoxicillin clavulanate (AC) is the most implicated drug in DILI worldwide. The clinical manifestations of amoxicillin clavulanate-induced liver injury (AC-DILI) are fatigue and jaundice, which some allergic features may accompany, but autoimmune phenomena are uncommon. Here, we describe a special case report of a patient with AC-DILI accompanied by autoimmune phenomena for the first time. The patient was a middle-aged Chinese woman who developed liver damage after taking AC for a period of time, with a RUCAM score of 6. The patient tested positive for antinuclear antibodies and had elevated levels of IgG. Human leukocyte antigen (HLA)-targeted sequencing results showed that the patient did not carry known AC-DILI-related HLA polymorphisms, but Sanger sequencing suggested that the patient had the ERAP2 rs1363907 mutation, which may be a pathogenic factor of AC-DILI in the patient. The patient’s progress notes, disease diagnosis, and treatment are summarized, and the role of ERAP2 pathogenic mutation rs1363907 in AC-DILI is discussed.

## Introduction

DILI is a type of liver damage caused by drugs and their metabolites, and the incidence ranges from 14/100,000 to 23.8/100,000 in the general population (Bjornsson et al., 2013; Sgro et al., 2002; Shen et al., 2019; Wang et al., 2022). DILI is typically classified as direct, idiosyncratic, or indirect (Hoofnagle and Bjornsson, 2019). Direct hepatotoxicity is a common, predictable, and dose-related injury that usually occurs immediately after exposure to high doses of the drug (Lucena et al., 2020). Indirect hepatotoxicity is mainly related to the indirect effect of drugs on the liver or immune system (Huffman et al., 2018). Idiosyncratic hepatotoxicity is an unpredictable, dose-independent DILI. It is rare, but when it does appear, the clinical phenotype is severe (Fontana et al., 2023). The underlying mechanism of direct and indirect hepatotoxicity is fully well understood, whereas that of idiosyncratic liver injury is not. Case–control studies suggest that genetic variation is one of the important reasons for susceptibility to DILI. Most genetic associations within the major histocompatibility complex (MHC) region of chromosome 6 are linked to human leukocyte antigen (HLA) class I and II alleles. A genetic variant is responsible for DILI in only one or a few specific agents, such as HLA-B*35:02 for minocycline (Urban et al., 2017), HLA-B*57:01 for flucloxacillin (Daly et al., 2009), and HLA-DQA1*02:01 for lapatinib (Spraggs et al., 2011). Therefore, there are still many idiosyncratic DILIs whose underlying causes have not been found.

The idiosyncratic injury occurs in a variety of drugs, among which AC is the most implicated drug in DILI worldwide. The clinical manifestations of AC-DILI are fatigue and jaundice, which some allergic features, including fever, rash, arthralgia, and eosinophilia, may accompany. However, a case of AC-DILI accompanied by autoimmune phenomena has not been reported yet. The identified risk factors for AC-DILI are HLA-A*02:01 (Lucena et al., 2011), HLA-DRB1*15:01 (Lucena et al., 2011; Stephens et al., 2013), HLA-DQB1*06:02 (Lucena et al., 2011), HLA-B*15:18 (Nicoletti et al., 2023), PTPN22 rs2476601 (Cirulli et al., 2019), and ERAP2 rs1363907 (Nicoletti et al., 2023), which were derived from large-scale genome-wide association studies in European and American populations.

Here, we report a female Chinese patient who was confirmed to have developed AC-DILI with autoimmune phenomena. She did not carry the HLA susceptibility allele, but EARP2 rs1363907 was detected. The role of ERAP2 rs1363907 in inducing AC-DILI in the Chinese population is highlighted. The uniqueness of this case and its importance for the diagnosis and prediction of AC-DILI are discussed.

## Case description

A 52-year-old Chinese woman went to the Department of Hepatology on 15 June 2023 with a 7-day history of yellow skin and abnormal liver function. The patient was conscious and in a good mental state. She had no symptoms such as abdominal distension, nausea, or fatigue. The patient’s skin and sclera were slightly yellowish, and urine became darker. The patient was diagnosed with breast cancer 3 years ago and underwent surgery and postoperative chemotherapy. Regular reinspection showed no recurrence. The patient has neither a history of chronic liver disease nor long-term alcohol consumption.

The patient reported that she went to another hospital 9 years ago due to fatigue, anorexia, and yellow urine. The diagnosis showed DILI, but the specific drug was unclear. On 31 January 2023, the patient was admitted to another hospital due to nausea, retching, and palpitation. Liver function tests showed liver damage (ALT: 2,145.7 U/L, AST: 1,235.5 U/L) (Table 1 **)**. Due to the lack of examination results, alkaline phosphatase (ALP), total bilirubin (TBiL), direct bilirubin (DBiL), and coagulation values are not available. After liver-protective treatment, the patient’s liver function returned to normal, and she was discharged. On 14 May 2023, the patient was admitted to another hospital due to fever, and the liver function results showed that the liver was damaged again (ALT: 275 U/L, AST: 259.2 U/L) (Table 1). Due to the lack of examination results, ALP, TBiL, DBiL, and coagulation values are unavailable. Subsequently, 1 week before the patient came to our hospital, that is, 8 June 2023, yellow urine and skin appeared again.

**TABLE 1:** Laboratory test results of patients from onset to recovery.

Investigation	Results														References
	2023											2024			
	Jan 31	May 14	Jun 8	Jun 17	Jun 24	Jul 7	Jul 21		Aug 22	Sep 19	Nov 21	Feb 16	May 14	Aug 13	
Liver function tests															
TP	—	—	—	66.18	63.38	77.61		72.93	69.32	72.6	69.2	73.05	77.6	70.59	65–85 g/L
ALB	—	—	—	34.77	32.77	40.63		39.25	39.3	39.57	39.26	42.72	44.7	43.85	35–55 g/L
GLB	—	—	—	31.41	30.61	36.98		33.68	30.02	33.03	29.94	30.33	32.9	26.74	20.0–40.0 g/L
TBil	—	—	—	56.07	29.85	22.86		14.99	13.00	12.52	13.22	11.45	13.8	11.5	5.1–20.0 μmol/L
DBil	—	—	—	36.13	21.35	12.29		5.95	3.55	2.93	3.07	2.73	2.98	2.86	0–6.10 μmol/L
I-Bil	—	—	—	19.94	8.5	10.57		9.04	9.45	9.59	10.15	8.72	10.82	-	5.10–20.0 μmol/L
A-G ratio	—	—	—	1.11	1.07	1.1		1.17	1.31	1.20	1.31	1.41	1.36	1.64	1.5–2.5 ng/mL
ALT	2,145.7	275	—	778.6	302.9	513.8		127	96.5	77.1	53.6	47	41.1	31.1	9–50 U/L
AST	1,235.5	259.2	—	832.4	220.8	400		60.7	70.4	52.4	49.1	40.2	39.7	36.8	15–40 U/L
ALT/AST	1.74	1.06	—	0.94	1.37	1.28		2.09	1.37	1.47	1.09	1.17	1.04	0.84	—
AST/ALT	0.58	0.94	—	1.07	0.73	0.78		60.7	72.95	0.68	0.92	0.86	0.97	1.18	—
TBA	—	—	—	43.47	—	4.63		5.24	2.05	3.56	—	1.63	—	3.16	0–25.0 μmol/L
ALP	—	—	—	178.1	—	143.1		100.3	64.6	60	55.5	54.5	85.8	75.9	50–135 U/L
Coagulation test															
PT	—	—	—	11.6	—	10.2		—	—	—	—	—	—	—	9.0–12.5 s
PTA	—	—	—	88.9	—	124.5		—	—	—	—	—	—	—	70%–130%
Quantitative FIB	—	—	—	1.94	—	2.12		—	—	—	—	—	—	—	2.00–4.00 g/L
APTT	—	—	—	27.6	—	24.7		—	—	—	—	—	—	—	25.0–34.0 s
TT	—	—	—	21	—	18.3		—	—	—	—	—	—	—	14.0–21.0 s
D-D quantification	—	—	—	0.61	—	—		—	—	—	—	—	—	—	0–0.55 mg/L
AT III activity assay	—	—	—	—	—	—		—	—	—	—	—	—	—	82%–132%
FDP	—	—	—	—	—	—		—	—	—	—	—	—	—	0–5 μg/mL
R value	14.7														
Injury phenotype	Hepatocellular type														

A-G ratio, albumin and globulin ratio; ALB, albumin; ALP, alkaline phosphatase; ALT, alanine aminotransferase; APTT, activated partial thromboplastin time; AST, aspartate aminotransferase; AT III activity assay, antithrombin III activity assay; DBil, direct bilirubin; D-D quantification, D-dimer quantification; FDP, fibrinogen degradation products; FIB, fibrinogen; GLB, globulin; I-Bil, indirect bilirubin; PT, prothrombin time; PTA, prothrombin activity; TBA, total bile acid; TBil, total bilirubin; TP, total protein; TT, thrombin time.

R value, R= (ALT/ULN) / (ALP/ULN). The R value is calculated based on the first available abnormal liver biochemical test results. Injury phenotype is distinguished according to R value: R ≥ 5 is hepatocellular, R ≤ 2 is cholestatic, and 2 < R < 5 is mixed.

The examination results during hospitalization showed that the patient’s liver function was impaired (ALT: 778.6 U/L, AST: 832.4 U/L, TBil: 56 μmol/L, DBil: 36.13 μmol/L, GGT: 430.6 U/L, ALP: 178.1 U/L) and her coagulation function was normal (PT: 11.6 s, PTA: 88.9%). The patient had no family history of liver disease, and her urine copper and serum ceruloplasmin levels were standard. A complete set of parasite tests, systemic lupus erythematosus, immunity checks, and IgG4 immunoglobulin levels were negative. Hepatitis A, B, C, D, E, Epstein‒Barr virus, and cytomegalovirus markers were negative. Routine blood tests and abdominal B-ultrasound results were normal. The patient’s autoantibodies were checked, including pANCA, cANCA, PR3-ANCA, MPO-ANCA, ANA, anti-dsDNA, anti-Sm, anti-nRNP, anti-SSA, anti-SSB, anti-histone; anti-rRNP; anti-Scl-70; anti-nucleosome; anti-Jo-1; anti-ribosomal P protein; AMA-M; anti-CENPB and anti-LKM-1. The results demonstrated that all antibodies were negative or within normal reference ranges, except for ANA (antinuclear antibody), which showed a positive result (nuclear-speckled pattern, 1:100 titer). The level of immunoglobulin G is close to the upper limit of the normal range (lgG: 16 g/L). The patient reported intermittent use of both traditional Chinese medicine (TCM) and amoxicillin clavulanate (AC) over the past 6 months. The prescription details for AC were as follows: amoxicillin and clavulanate potassium tablets (containing amoxicillin 0.25 g and clavulanate potassium 0.125 g per tablet), administered orally as one tablet three times daily. The patient had been taking AC continuously for 1 week before symptom onset. As the patient could not furnish details regarding the TCM used, the exact composition remains untraceable. We performed RUCAM scoring for AC, yielding a score of 6, which indicates a probable likelihood of DILI (Danan and Teschke, 2015). However, as the specific details of TCM use were unclear, the potential influence of TCM requires further investigation. We recommended that the patient stop taking TCM and AC and provided her with symptomatic and supportive treatments simultaneously. The specific regimen was magnesium isoglycyrrhizinate injection, once daily, 0.1 g each time, dilute with 10% dextrose injection 250 mL and then dripped intravenously, which is used to protect the liver and improve abnormal liver function; acetylcysteine injection, once daily, 8 g each time, diluted in 250 mL of 10% glucose injection for intravenous drip, for reducing bilirubin levels, improving prothrombin activity, and preventing liver failure; hepatocyte growth-promoting factors enteric-coated capsules (orally, 100 mg–150 mg three times daily) to promote hepatocyte regeneration. The above regimen was used continuously for 1 week. The patient improved and was discharged after a week. We recommended that the patient take diammonium glycyrrhizinate enteric-coated capsules (orally, 150 mg three times daily) for 2 weeks after discharge and return for follow-up in 14 days.

**FIGURE 1:**
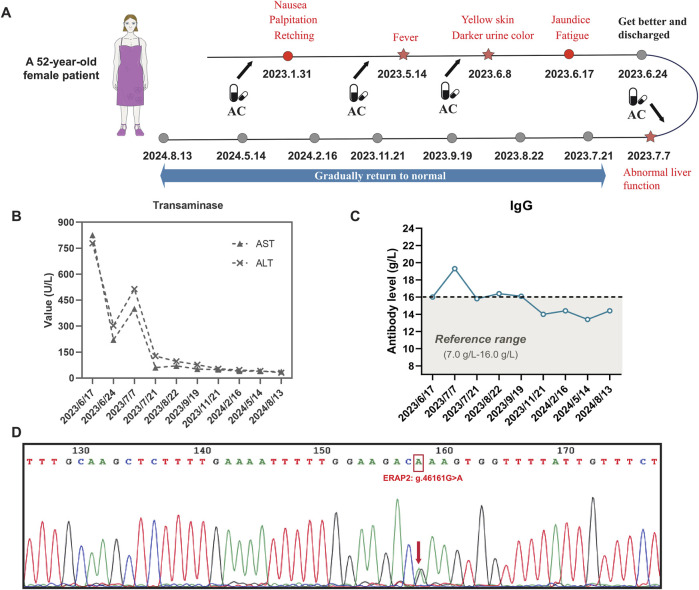
AC-DILI timeline and patient laboratory test results. **(A)** Timeline from AC-DILI onset to recovery. The arrow represents the time point of taking AC, and the star represents the time of relapse; **(B)** transaminase levels from admission to recovery; **(C)** IgG antibody levels from admission to recovery; **(D)** Sanger sequencing result. The patient carried the g.46161G>A intron variant (rs1363907) in the ERAP2 gene. The g.46161G>A mutation is an eQTL that regulates the expression of ERAP2.

Two weeks after discharge, the patient returned to the hospital for a follow-up examination, and it found that aspartate aminotransferase and alanine aminotransferase were elevated again (ALT: 513.8 U/L, AST: 400 U/L), antinuclear antibodies were positive, and the IgG level increased to 19.3 g/L. These results indicated that the patient had liver injury again accompanied by autoimmune characteristics. The patient no longer took TCM but did take AC. The patient took AC for upper respiratory infection symptoms, including runny nose, sneezing, and sore throat. The specific medication details are as follows: amoxicillin and clavulanate potassium tablets (amoxicillin 0.25 g + clavulanate potassium 0.125 g per tablet), orally administered, one tablet three times daily. The medication was continued for 3 consecutive days and discontinued on the day of scheduled hospital follow-up. No other medications were taken during this period. After admission, she underwent routine blood and nucleic acid tests, and the upper respiratory infection symptoms were found to be the result of a SARS-CoV-2 infection. The typical symptoms of SARS-CoV-2 infection include fever, fatigue, and upper respiratory symptoms. While some patients may develop liver injury, it generally manifests as a mild elevation of liver enzymes, which is associated with systemic inflammatory response or direct viral injury. The typical manifestations of DILI include fatigue, nausea, and jaundice (in severe cases), accompanied by a clear history of medication exposure. Laboratory findings typically show marked elevation of liver enzymes (often >5×ULN), which may be associated with increased bilirubin levels. Although the patient in this case report had a concomitant SARS-CoV-2 infection, the presence of a well-documented drug exposure history and significantly elevated liver enzymes is consistent with the diagnostic features of drug-induced liver injury (DILI). A review of the patient’s medication history revealed that her previous episodes of liver injury were roughly consistent with the time she was taking AC. Based on the above information, it was determined that the patient’s recurrent liver injury was AC-DILI (Figures 1A–C).

Each time the patient took AC, the liver was damaged and eventually developed autoimmune features. To investigate disease susceptibility, we employed HLA-targeted sequencing to assess whether patients carried pathogenic HLA polymorphisms. Our sequencing comprehensively covered all loci within the HLA genetic region, enabling complete identification of all HLA alleles in patients and investigation of potential HLA alleles associated with AC-DILI. The sequencing was performed on the Illumina NovaSeq6000 platform, achieving 100× sequencing depth with 99% coverage. Table 2 presents detailed results, showing that the patient did not carry any known HLA alleles associated with AC-DILI. We used Sanger sequencing to detect non-HLA variants of AC-DILI, PTPN22 rs2476601, and ERAP2 rs1363907 and found that the patient carried ERAP2 rs1363907 (Figure 1D). An ERAP2 protein is an aminopeptidase found in the endoplasmic reticulum of all cells and is involved in trimming antigenic peptides presented by MHC-I. A transcriptome-wide association study and a genome-wide association study based on 444 AC-DILI cases and 10,397 controls revealed a significant association of ERAP2 rs1363907 with AC-DILI risk (Nicoletti et al., 2023). In addition, previous studies have shown that genetic variations of ERAP2 are associated with various autoimmune diseases, such as spondylitis (Hanson et al., 2018; Tsui et al., 2010) and psoriasis (Zhen et al., 2019; Marusina et al., 2023). Therefore, we speculate that ERAP2 rs1363907 may increase susceptibility to AC-DILI and trigger autoimmune phenomena.

**TABLE 2:** HLA-targeted sequencing reveals HLA alleles carried by the patient.

Gene	Classification	Allele1	Allele2
HLA-A	Class Ⅰ	HLA-A*02:06:01	HLA-A*02:03:01
HLA-B	Class Ⅰ	HLA-B*35:01:01	HLA-B*38:02:01
HLA-C	Class Ⅰ	HLA-C*07:02:01	HLA-C*03:03:01
HLA-DMA	Class Ⅱ	HLA-DMA*01:01:01	Not typed
HLA-DMB	Class Ⅱ	HLA-DMB*01:01:01	Not typed
HLA-DOA	Class Ⅱ	HLA-DOA*01:01:01	Not typed
HLA-DOB	Class Ⅱ	HLA-DOB*01:01:01	Not typed
HLA-DPA1	Class Ⅱ	HLA-DPA1*02:02:02	Not typed
HLA-DPA2	Class Ⅱ	HLA-DPA2*01:01:02	HLA-DPA2*01:01:01
HLA-DPB1	Class Ⅱ	HLA-DPB1*05:01:01	Not typed
HLA-DQA1	Class Ⅱ	HLA-DQA1*01:02:02	HLA-DQA1*03:02:01
HLA-DQB1	Class Ⅱ	HLA-DQB1*05:02:01	HLA-DQB1*03:03:02
HLA-DRA	Class Ⅱ	HLA-DRA*01:01:01	Not typed
HLA-DRB1	Class Ⅱ	HLA-DRB1*16:02:01	HLA-DRB1*09:01:02
HLA-DRB2	Class Ⅱ	Not typed	Not typed
HLA-DRB3	Class Ⅱ	Not typed	Not typed
HLA-DRB4	Class Ⅱ	HLA-DRB4*01:03:01	Not typed
HLA-DRB5	Class Ⅱ	HLA-DRB5*01:01:01	HLA-DRB5*01:22:01
HLA-DRB6	Class Ⅱ	Not typed	Not typed
HLA-DRB7	Class Ⅱ	HLA-DRB7*01:01:01	Not typed
HLA-DRB8	Class Ⅱ	HLA-DRB8*01:01	Not typed
HLA-DRB9	Class Ⅱ	Not typed	Not typed
HLA-E	Class Ⅰ	HLA-E*01:03:01	HLA-E*01:01:01
HLA-F	Class Ⅰ	HLA-F*01:01:01	Not typed
HLA-G	Class Ⅰ	HLA-G*01:01:01	Not typed
HLA-H	Class Ⅰ	HLA-H*01:01:01	Not typed
HLA-J	Class Ⅰ	HLA-J*01:01:01	Not typed
HLA-K	Class Ⅰ	HLA-K*01:02	Not typed
HLA-L	Class Ⅰ	HLA-L*01:01:01	HLA-L*01:02
HLA-T	Class Ⅰ	HLA-T*01:01:01	HLA-T*01:01:01
HLA-V	Class Ⅰ	HLA-V*01:01:01	Not typed
HLA-W	Class Ⅰ	HLA-W*03:01:01	Not typed
HLA-Y	Class Ⅰ	Not typed	Not typed

After discharge, the patient continued to use diammonium glycyrrhizinate enteric-coated capsules to protect her liver. Because her IgG antibody and antinuclear antibody levels were still outside the normal range, it was recommended that she take methylprednisolone tablets to improve her symptoms. Regular follow-up examinations were necessary until the liver function returned to normal (Table 1). Based on clinical diagnosis and genetic testing results, it was recommended that the patients should not take AC. The 1-year follow-up showed that the patient had no symptoms, such as yellow skin or dark yellow urine. The enzyme indicators and IgG level had completely returned to normal values. In addition, the patient has not taken AC according to the prompts of ERAP2 rs1363907 and has not experienced liver damage so far.

## Discussion

DILI is an important adverse drug reaction (ADR), which can lead to acute liver failure or even death in severe cases. The diagnosis of DILI currently remains an exclusive strategy. Therefore, a detailed medical history and careful exclusion of other potential causes of liver injury are key to establishing a correct diagnosis (Andrade et al., 2019; Aithal et al., 2011). DILI can be non-idiosyncratic or idiosyncratic (Leise et al., 2014). Non-idiosyncratic injury is divided into direct hepatotoxicity and indirect hepatotoxicity. The former is closely related to drug dosage, and the classic drug is acetaminophen, which can easily lead to fulminant liver failure after overdose (Grudzinski et al., 2019). The latter echoes the effects of the drug. For example, the hepatotoxicity of immune checkpoint inhibitors results from the blocking of inhibitory factors of the immune system (De Martin et al., 2020). In contrast, idiosyncratic hepatotoxicity is erratic. Gender (Chalasani et al., 2008), age (Abboud and Kaplowitz, 2007), underlying liver disease (Wong et al., 2000), HIV infection (Tseng and Wong, 2016), obesity (Ma et al., 2024), and genetic variations (Kaliyaperumal et al., 2018) are generally considered risk factors, with genetic variation being the least controversial. Genetic variations of targets related to drug metabolism, HLA, cytokines, oxidative stress, and hepatobiliary transporters are reasonable explanations for the occurrence of DILI with specific drugs (Kullak-Ublick et al., 2017). Drugs that cause idiosyncratic liver injury vary in different countries and regions, but AC is the most common agent causing DILI in both European and Asian populations.

AC combines amoxicillin (a semi-synthetic antimicrobial agent) and clavulanic acid (a β-lactamase inhibitor). AC shows good activity against various bacteria and is the most consumed β-lactam antibiotic worldwide (Easton et al., 2003). DILI is one of the concerning adverse reactions of AC. There is a latency period between taking the medicine and the onset of symptoms, usually 3–90 days. The typical pattern of AC-DILI is cholestatic injury, but it can also be hepatocellular (Chalasani et al., 2014). In this case, the patient had hepatocellular AC-DILI.

The pathogenesis is unknown, but AC-DILI is an immune-mediated ADR formed by immune cells attacking bile duct cells or liver cells (Karnes et al., 2019). Genetic studies suggest that some genetic variants are responsible for susceptibility to AC-DILI, most located in the HLA region (Aithal and Grove, 2015). HLA molecules play a crucial role in antigen presentation and T cell activation, distinguishing self/non-self by presenting thousands of peptides to the T cell receptor (TCR). During the occurrence of ADRs, the drug is the “peptide” displayed. HLA molecules present drugs to TCR through three modes: the hapten model, the pharmacological interaction model, and the altered repertoire model. AC belongs to the typical hapten model; that is, AC forms an irreversible covalent bond with the protein, and then the drug-peptide adduct is combined into the mutated HLA molecule pocket and is finally presented to the TCR to activate the T cell (Tailor et al., 2020; Jaruthamsophon et al., 2022). HLA polymorphisms associated with AC-DILI include HLA-A*02:01, HLA-DRB1*15:01, HLA-B*15:18, and HLA-DQB1*06:02. With the patient’s consent, we collected blood samples to extract DNA for HLA-targeted sequencing. The results showed that the patient did not carry any of the above-mentioned pathogenic polymorphisms (Table 2). It is worth noting that HLA polymorphisms conferring susceptibility to AC-DILI were identified in large-scale genome-wide association studies, and the samples were from European and American populations. No relevant studies have been reported on the Chinese population, so further exploring the AC-DILI pathogenic polymorphisms specific to the Chinese population is necessary.

In addition to HLA polymorphisms, two non-HLA gene mutations are strongly associated with the risk of AC-DILI: PTPN22 rs2476601 and ERAP2 rs1363907. Sanger sequencing results showed that PTPN22 rs2476601 was not detected in the patient, but ERAP2 rs1363907 existed. ERAP2 is an endoplasmic reticulum aminopeptidase in the same family as ERAP1, and both belong to the zinc m1 family metallopeptidase. ERAP2 and ERAP1 act in concert to trim and present peptides that bind to HLA class I molecules within the endoplasmic reticulum (Haroon and Inman, 2010). It has been reported that ERAP2 may over-trim some peptides, so they cannot bind HLA molecules (de Castro and Stratikos, 2019). Therefore, it can be speculated that the hapten formed by covalent binding of AC to endogenous peptides will be over-trimmed by ERAP2, which is typically expressed and performs protein functions, to form short peptides that cannot bind to HLA class I molecules. ERAP2 rs1363907 is an expression quantitative trait locus (eQTL) affecting the expression of ERAP2. Thus, the trimming of the hapten peptide is completed by ERAP1. The trimmed peptide will fit into the binding pocket of HLA class I molecules. HLA class I molecules present hapten peptides to the TCR, which cannot recognize dissidents and subsequently activates T cells to exert a killing effect. This may be the mechanism by which ERAP2 rs1363907 induces AC-DILI.

Notably, the patient is a middle-aged woman, which prompts the need to exclude autoimmune diseases. Autoimmune diseases are a group of disorders caused by an abnormal immune response targeting the body’s tissues. Most autoimmune diseases are more prevalent in women, particularly autoimmune hepatitis (AIH), thyroid disorders, systemic lupus erythematosus (SLE), and systemic sclerosis (SSc), which exhibit the highest female-to-male incidence ratios (Conrad et al., 2023). The differential diagnosis of autoimmune hepatitis (AIH) includes elevated liver enzymes, positive autoantibodies (including ANA, anti-SMA, and anti-LKM1), and liver biopsy showing interface hepatitis. Thyroid disorders are characterized by thyroid dysfunction and specific autoantibodies (including TPOAb, TgAb, and TRAb). Systemic lupus erythematosus (SLE) presents with multi-system clinical manifestations, positive autoantibodies (including high-titer ANA, anti-dsDNA, and anti-Sm), and decreased complement levels. Systemic sclerosis (SSc) is characterized by skin sclerosis, Raynaud’s phenomenon, and positive anti-Scl-70 antibody. In this case, the patient demonstrates no features of thyroid disease, SLE, or SSc. Although exhibiting some characteristics of AIH (positive ANA and elevated IgG levels), the patient does not meet the diagnostic criteria for AIH. Therefore, the patient presents with autoimmune phenomena but has not been diagnosed with any definitive autoimmune disorder.

The differential diagnosis of DILI requires the exclusion of other diseases that may cause similar liver injury manifestations. In this case, viral hepatitis, non-alcoholic fatty liver disease, alcoholic liver disease, biliary disorders, and inherited metabolic liver diseases were ruled out based on viral serology, copper metabolism tests, iron metabolism tests, and autoantibody testing. The patient had a clear history of AC use, followed by clinical manifestations, including elevated liver enzymes, fatigue, loss of appetite, and dark urine, all of which resolved after drug cessation. Additionally, genetic testing identified AC-DILI susceptibility variants. Collectively, these findings support the diagnosis of AC-DILI. However, several limitations remain. First, the lack of liver biopsy results precludes definitive exclusion of AIH. The patient tested positive for ANA with elevated IgG levels, which partially overlap with characteristic features of AIH. Second, the patient had a history of concomitant use of traditional Chinese medicines during episodes of liver injury. Incomplete documentation of medication details hinders definitive assessment of potential confounding effects from other agents. In conclusion, the nonspecific clinical features of DILI necessitate a diagnostic approach integrating medication history, laboratory findings, imaging studies, and exclusion of alternative etiologies. Early recognition and prompt drug discontinuation remain critical to clinical management.

From the patient’s perspective, the mutation detection and medication recommendations based on the results will help prevent potential DILI. This will provide the patient with a reference for long-term safe medication use. Although ERAP2 rs1363907 was ultimately identified as a possible disease trigger, its molecular mechanism must still be proved by *in vivo* and *in vitro* experiments.

## Data Availability

The datasets presented in this study can be found in online repositories. The names of the repository/repositories and accession number(s) can be found below: https://www.ncbi.nlm.nih.gov/, PRJNA1190665.
